# Is the National Health Insurance Scheme helping pregnant women in accessing health services? Analysis of the 2014 Ghana demographic and Health survey

**DOI:** 10.1186/s12884-021-03651-6

**Published:** 2021-03-12

**Authors:** Edward Kwabena Ameyaw, Bright Opoku Ahinkorah, Linus Baatiema, Abdul-Aziz Seidu

**Affiliations:** 1 School of Public Health, Faculty of Health, University of Technology Sydney, PMB University Private Mail Bag, Sydney, NSW Australia; 2 Department of Population and Health, Faculty of Social Sciences, College of Humanities and Legal Studies, University of Cape Coast, Cape Coast, Ghana; 3 College of Public Health, Medical and Veterinary Sciences, James Cook University, Townsville, Queensland Australia

**Keywords:** National Health Insurance Scheme, Health service, Pregnant women, Maternal healthcare, Ghana

## Abstract

**Background:**

Increasing the use of healthcare is a significant step in improving health outcomes in both the short and long term. However, the degree of the relationship between utilization of health services and health outcomes is affected by the quality of the services rendered, the timeliness of treatment and follow-up care. In this study, we investigated whether the National Health Insurance Scheme (NHIS) is helping pregnant women in accessing health services in Ghana.

**Methods:**

Data for the study were obtained from the women’s file of the 2014 Ghana Demographic and Health Survey. All women with birth history and aged 15–49  constituted our sample (*n* = 4271). We employed binary logistic regression analysis in investigating whether the NHIS was helping pregnant women in accessing health service. Statistical significance was set at <0.05.

**Results:**

Most women had subscribed to the NHIS [67.0%]. Of the subscribed women, 78.2% indicated that the NHIS is helping pregnant women in accessing healthcare. Women who had subscribed to the NHIS were more likely to report that it is helping pregnant women in accessing health service [aOR = 1.70, CI = 1.38–2.10]. We further noted that women who had at least four antenatal visits were more likely to indicate that NHIS is helping pregnant women in accessing health services [aOR = 3.01, CI = 2.20–4.14]. Women with secondary level of education [aOR= 1.42; CI: 1.04–1.92] and those in the richest wealth quintile [aOR = 3.51; CI = 1.94–6.34] had higher odds of indicating that NHIS is helping pregnant women in accessing healthcare. However, women aged 45–49 [aOR = 0.49; CI = 0.26–0.94], women in the Greater Accra [aOR = 0.29; CI = 0.16–0.53], Eastern [aOR = 0.12; CI = 0.07–0.21], Northern [aOR = 0.29; CI = 0.12–0.66] and Upper East [aOR = 0.17; CI = 0.09–0.31] regions had lower odds of reporting that NHIS is helping pregnant women in accessing health services.

**Conclusion:**

To enhance positive perception towards the use of health services among pregnant women, non-subscribers need to be encouraged to enrol on the NHIS. Together with non-governmental organizations dedicated to maternal and child health issues, the Ghana Health Service’s Maternal and Child Health Unit could strengthen efforts to educate pregnant women on the importance of NHIS in maternity care.

## Background

An important step in improving health outcomes in both the short and long term is increasing healthcare access [1]. The magnitude of the association between the use of health services and health outcomes, however, is influenced by the quality of the services provided, the timeliness of receiving treatment and follow-up care [2]. As stipulated in the Sustainable Development Goal (SDG) 3, achieving universal health coverage has become a global agenda. This is to ensure that in times of need, all persons have access to quality health services and are shielded from the financial burdens of health care expenses [3]. In recent decades, several low-and middle-income countries (LMICs) have made substantial efforts to achieve this objective by introducing a number of Social Health Protection (SHP) pre-payment systems that seek to minimize dependency on out-of-pocket payments. Ghana has emerged in sub-Saharan Africa as a leader of these health financing reforms, being the first country in the sub-region to introduce a National Health Insurance Scheme (NHIS) [4, 5].

The Government of Ghana launched the NHIS-an initiative to provide health insurance to everyone by removing the out-of-pocket payment hurdle in 2003 and this became fully operational in 2005 [5, 6]. Health care services were paid for predominantly via consumer fees (also referred to as cash-and-carry) prior to the implementation of this program but this arrangement disenfranchised the impoverished and disadvantaged from accessing healthcare [7, 8]. The NHIS Act 852 (2012) urges all Ghanaians to enrol unto the health insurance scheme [9]. Funding for the scheme mainly comes from a 2.5% national tax on goods and services and a social security tax on formal workers [10]. Personal insurance premiums are relatively low, about US$10 per year in 2010 [11]. In fact, all nationals must register for insurance, although those who do not register will not be punished. For certain vulnerable groups, including the elderly (defined as those over 70), people under 18, and pregnant women and their newborns, the NHIS is provided free of charge.

Considering the various pregnancy-related complications experienced by pregnant women during pregnancy, tax exemption for pregnant women’s health insurance premiums has become crucial. These include high blood pressure, severe persistent nausea, vomiting and gestational diabetes [12]. Dealing with these complications will impose an economic burden on pregnant women in LMICs such as Ghana, which can lead to serious health complications. Despite the NHIS premium exemptions for pregnant women, unavailability of health facilities, travel cost to health facilities, perceived quality of service, travel cost to NHIS registration centres and socio-cultural factors have been found to be associated with low enrollment [13]. It is also found that the quality of health services provided by health institutions is poor, and factors such as long waiting times, poor attitudes of health institution staff, and shortage of drugs, are all contributing to low enrollment rate of NHIS [11, 14].

Various studies have been conducted on NHIS in Ghana. Some of these have focused on subscribers’ perception [5, 15, 16] and determinants of enrolment [6, 17–22]. Others have also investigated the retention of subscribers [23], reducing medical claims cost [24], sustainability of the scheme [25], equity in accessibility [26–28], variation in coverage [29], quality healthcare assessment [30] and trends in subscription [13]. Despite comprehensive research on the NHIS, none of these studies have, to the best of our knowledge, focused on the position of women as to whether NHIS is helping pregnant women in accessing health services or not. It is against this background and the gap in the literature that this study seeks to test the hypothesis that NHIS is helping pregnant women in accessing health services by using the current nationally representative data–2014 Ghana Demographic and Health Survey (GDHS). Outcome of this study is anticipated to inform and strengthen the need for pregnant women to subscribe to the NHIS which can help in the reduction of maternal mortality cases, improve maternal and child health and contribute to the attainment of the SDG 3.7.

## Methods

### Data source

We used data from the women recode file of the 2014 GDHS. This is the sixth version since the survey started in Ghana in 1988. It forms part of the Measure DHS Program which seeks to monitor core health indicators in LMICs. Two stage sample design was carried out. The initial stage involved the selection of 427 clusters constituting the enumeration areas (EAs). The enumeration areas emerged from urban (216) and rural (211) locations across all the ten regions at the time. The second phase involved the selection of 11,835 households from the EAs and this resulted in a total sample of 9396 women aged 15–49. The survey had 97.3% response rate [31]. For the purpose of our study, 4271 women with complete data were included.

### Dependent variable

The dependent variable was whether the NHIS is helping pregnant women for health services or not. The question was posed to women aged 15–49 during the 2014 GDHS. It was accompanied by two responses: “Yes” and “No”. This variable was chosen on the premise that one of the priorities of the pro-poor NHIS in Ghana is to ease the financial burden in accessing maternity services [32]. As a result, investigating the perception of women on whether this mandate is being achieved is essential for future health financing policy directions.

### Independent variables

Eight independent variables were included in this study. Of these, the main independent variable was health insurance subscription. The other included variables were Age (15–19,20-24,25-29,30–34-35-39,40-44,45–49), education (No education, primary, secondary, tertiary), residence (rural,urban), antenatal care (ANC) visits (Below 4 Visits, At least 4 Visits), current pregnancy status (pregnant, not pregnant) and region (Western, Central, Greater Accra, Volta, Eastern, Ashanti, Brong Ahafo, Northern, Upper East, Upper West) and wealth quintile (poorest, poorer, middle, richer, richest). Wealth, in the DHS, is a composite measure computed by combining data on a household’s ownership of carefully identified assets including television, bicycle, materials used for house construction, sanitation facilities and type of water access. Principal component analysis was used to transform these variables into wealth index by placing individual households on a continuous measure of relative wealth. The DHS segregates households into five wealth quintiles; poorest, poorer, middle, richer and richest. These variables have been reported as essential for investigating NHIS [33, 34].

### Statistical analyses

Stata version 13 was used to analyse the data using both descriptive and inferential statistics. In our descriptive analysis, we computed the proportion of women in each of the aforementioned independent variables. The proportion of women who indicated either “Yes” or “No” on whether the NHIS is helping pregnant women in health services was also calculated (see Table 1). Chi-square tests were conducted in order to ascertain the independent variables that had significant association with the dependent variable. With the exception of “current pregnancy status”, all the independent variables were significant and were included in our inferential analysis, where three Binary Logistic Regression models were fitted in all. This analytical approach was the most suitable option premised on the fact that our dependent variable had two outcomes. The first model (Model I) accounted for NHIS subscription and whether it helps pregnant women in accessing health services. In model two, we adjusted for the effect of ANC visit-as a woman needs to first access healthcare during pregnancy in order to know whether the NHIS helps in healthcare during pregnancy or otherwise. All the seven significant independent variables were fitted in the final model (Model III) after which post-estimation test (Linktest) was conducted to determine whether the model is devoid of model specification error and also to ensure that relevant variables have not been omitted. Multicollinearity was also checked and we found no evidence of multicollinearity. Results for Model I was reported as odds ratio (OR) whilst that of Model II and III were reported as adjusted odds ratios (aOR) with their respective confidence intervals which were considered statically significant at 95%. Samples were weighted to adjust for the sample design.

**Table 1:** Socio-demographic characteristics of women (*N* = 4271)

Variable	Weighted N	Percentage %	NHIS helping pregnant women for health services		X^**2**^ (***p*** value)
**Health Insurance Subscription**			**Yes**	**No**	81.7(*p* < 0.001)
Subscribed	2860	67.0	78.2	21.8	
Unsubscribed	1411	33.0	65.0	35.0	
**Age**					23.6(*p* < 0.001)
15–19	190	4.5	76.0	24.0	
20–24	727	17.0	73.8	26.2	
25–29	1032	24.2	75.5	24.5	
30–34	1001	23.4	24.2	75.8	
35–39	806	18.9	24.5	75.5	
40–44	390	9.1	31.8	68.2	
45–49	125	2.9	38.8	61.2	
**Education**					122.6(*p* < 0.001)
No education	1109	26.0	64.6	35.4	
Primary	835	19.6	72.8	27.2	
Secondary	2129	49.8	81.0	19.0	
Higher	198	4.6	85.2	14.8	
**Wealth Quintile**					183.9(*p* < 0.001)
Poorest	895	21.0	61.5	38.5	
Poorer	863	20.2	73.8	26.2	
Middle	852	19.9	80.1	19.9	
Richer	842	19.7	82.7	17.3	
Richest	819	19.2	85.1	14.9	
**Residence**					25.8(*p* < 0.001)
Urban	1771	41.5	78.2	21.8	
Rural	2500	58.5	71.2	28.8	
**ANC Visits**					130.0(*p* < 0.001)
Below 4 Visits	525	12.3	54.8	45.2	
At least 4 Visits	3746	87.7	77.1	22.9	
**Current Pregnancy Status**					1.1 (0.304)
Pregnant	422	9.9	76.2	23.8	
Not pregnant	3849	90.1	73.9	26.1	
**Region**					595.5(*p* < 0.001)
Western	443	10.4	88.2	11.8	
Central	470	11.0	79.9	20.1	
Greater Accra	698	16.3	76.8	23.2	
Volta	326	7.6	78.3	21.7	
Eastern	401	9.4	50.8	49.2	
Ashanti	754	17.7	85.5	14.5	
Brong Ahafo	386	9.0	92.0	8.0	
Northern	496	11.6	54.3	45.7	
Upper East	182	4.3	50.9	49.1	
Upper West	115	2.7	93.4	6.6	

Source: 2014 GDHS

## Results

### Socio-demographic characteristics of women

Table 1 presents the results on background characteristics and whether NHIS is helping pregnant women to access health services. Twenty four percent of the women were aged 25–29 years. Nearly half of the women (49.8%) had attained secondary level of education with 21.0% of them within the poorest wealth quintile. More than half (58.5%) of the women were found within the rural settings. Seventeen percent were in the Ashanti region, 16.3% in Greater Accra, 11.6% in Northern and 2.7% in Upper West region. The majority (87.7%) of the women made at least 4 visits. Ninety percent (90.1%) of the women were not pregnant at the time of the survey and 67.0% were subscribers of NHIS. Approximately 18% of these women were found in Ashanti region. The chi-square test showed that age (X^2^ = 23.6, *p* < 0.001), education (X^2^ = 122.6, *p* < 0.001), wealth quintile (X^2^ = 183.9, *p* < 0.001), residence (25.8, *p* < 0.001), ANC visits (X^2^ = 130, *p* < 0.001), NHIS subscription (X^2^ = 81.7, *p* < 0.001) and region (X^2^ = 595.5, *p* < 0.001) had statistically significant association with the perception that NHIS helps pregnant women to access health services (see Table 1).

### Logistic regression results

Table 2 presents the logistic regression results on whether the NHIS is helping pregnant women to access health services or not. It was found that women who had subscribed to the NHIS had higher odds [aOR = 1.70; CI = 1.38–2.10] of indicating that NHIS is helping pregnant women in accessing health services compared to those who are not subscribed. The study showed that women who accessed ANC at least four times had higher odds [aOR = 1.99; CI = 1.43–2.77] to report that NHIS is helping to seek health services as compared to their counterparts who attained less than four ANC visits. Women with secondary level of education [aOR = 1.42; CI: 1.04–1.92] and those in the richest wealth quintile [OR = 3.51; CI = 1.94–6.34] had higher odds of indicating that NHIS is helping pregnant women in accessing healthcare. However, women aged 45–49 [aOR = 0.49; CI = 0.26–0.94], women in the Greater Accra [aOR = 0.29; CI = 0.16–0.53], Eastern region [aOR = 0.12; CI = 0.07–0.21], Northern [aOR = 0.29; CI = 0.12–0.66] and Upper East [aOR = 0.17; CI = 0.09–0.31] had lower odds of reporting that NHIS is helping them to access health services (see Table 2).

**Table 2:** Binary Logistic Regression analysis on whether NHIS is helping pregnant women access health services or not

Variable	Model I		Model II		Model III	
	OR 95% CI		aOR 95% CI		aOR 95% CI	
**Health Insurance Subscription**						
Unsubscribed	1	Reference	1	Reference	1	Reference
Subscribed	1.80***	[1.47–2.20]	1.61***	[1.32–1.96]	1.70***	[1.38–2.10]
**ANC Visits**						
Below 4 Visits			1	Reference	1	Reference
At least 4 Visits			3.01***	[2.20–4.14]	1.99***	[1.43–2.77]
**Age**						
15–19					1	Reference
20–24					0.88	[0.53–1.45]
25–29					0.76	[0.47–1.24]
30–34					0.79	[0.47–1.33]
35–39					0.77	[0.45–1.31]
40–44					0.56	[0.31–1.03]
45–49					0.49*	[0.26–0.94]
**Education**						
No education					1	Reference
Primary					1.23	[0.90–1.67]
Secondary					1.42*	[1.04–1.92]
Higher					1.48	[0.74–2.95]
**Wealth Quintile**						
Poorest					1	Reference
Poorer					1.23	[0.85–1.79]
Middle					1.98**	[1.34–2.95]
Richer					2.56***	[1.57–4.19]
Richest					3.51***	[1.94–6.34]
**Residence**						
Urban					1	Reference
Rural					1.46	[1.00–2.12]
**Region**						
Western					1	Reference
Central					0.72	[0.35–1.46]
Greater Accra					0.29***	[0.16–0.53]
Volta					0.57	[0.29–1.09]
Eastern					0.12***	[0.07–0.21]
Ashanti					0.71	[0.41–1.22]
Brong Ahafo					1.80	[0.93–3.52]
Northern					0.29**	[0.12–0.66]
Upper East					0.17***	[0.09–0.31]
Upper West					1.82	[0.60–5.52]
Linktest						
_hat						0.000
_hatsq						0.364
N						4721

Source: 2014 GDHS, *OR* Odds Ratio, *AOR* Adjusted Odds Ratio, *CI* Confidence Interval in brackets; 1 = reference; *p < 0.05, **p < 0.01, ***p < 0.001

## Discussion

The purpose of the study was to examine whether NHIS helps women to access health services during pregnancy. We found that in addition to NHIS subscription, ANC visits, age, educational level, wealth status and region influenced women’s perception of the importance of NHIS in healthcare delivery in Ghana. In terms of NHIS subscription, we found that women who had subscribed to NHIS were more likely to mention that NHIS is helping pregnant women to access health services. This confirms that NHIS is heping women to access health services. The findings support the findings of Singh et al. [35] who carried out a study on NHIS and maternal and child health and found that pregnant women who subscribed for NHIS were more likely to consider it as important for maternal healthcare delivery. Mensah et al. [36] and Dzakpasu et al. [37] have already argued that the NHIS have enhanced use of maternal care in some regions of Ghana. Similarly, Dixon et al. [38] identified that women who registered in the NHIS make more ANC visits relative to those who aren’t registered, irrespective of socio-economic and demographic characteristics. In support of the results, Duku et al. [39] reported that the NHIS in Ghana has an exemption policy as part of its efforts to protect vulnerable populations, which relieves them, including pregnant women, from paying an annual premium. As such, many women would use maternal health care services by minimizing the financial barriers to maternal care through NHIS. The result also indicates that NHIS subscription may indeed work as a pro-poor strategy in health financing by increasing access to health care.

The study found that women who accessed ANC at least 4 times had higher odds to report that NHIS is helping pregnant women to seek health services. Although this is a cross-sectional data, this reaffirms that the NHIS is really helping pregnant women in accessing health services. The finding confirms the findings of previous studies by Dalinjong, Wang and Homer [40] and Singh et al. [35], who explained that in the absence of delays in providing healthcare to pregnant women, they are more likely to utilize ANC and be satisfied with using NHIS.

Women with secondary level of education and those in the richest wealth quintile had higher odds of indicating that NHIS is helping pregnant women to access health services. The finding explains the link between empowerment and easy access to healthcare. A previous study by Nasrabadi, Sabzevari and Bonabi [41] has shown that persons who are empowered exhibit positive health outcomes, such as increased power of decision-making, freedom for making choices and responsibility acceptance, developing trust in relations, informed choice, hopefulness, speedy personal development, awareness of one’s own world, identification of one’s own strengths and abilities, feeling more powerful, higher self-confidence, higher self-efficacy, and eventually, an improved quality of life [41].

The findings also revealed regional-level heterogeneity in the perception that NHIS is helping pregnant women access healthcare. This is characterised by significant inequities in favour of the south [42] and these have influence on satisfaction with health services. Interestingly, pregnant women in two regions in the southern sector (Greater Accra and Eastern) and two regions in the Northern portion of the country (Northern and Upper East) showed lower odds of reporting that NHIS is helping them to access health services. Nevertheless, a previous study by Amo-Adjei et al. [43] found that women generally had better perceptions of the quality of NHIS healthcare in northern regions (Northern, Upper East and West) compared to those in other parts of the country. This could be due to differences in accessibility and higher quality of care experience, especially in the private sector compared to the public sector [44], which has a lower probability of being situated in the northern part of Ghana. Our finding implies that regional disparity in perception of the quality does not always lead to a positive perception towards the use of NHIS for health services. We therefore argue that the regional-level heterogeneity in perception towards the use of health services under the NHIS could be explained by other factors which can be explored in further studies.

### Strength and limitations

The study’s strength lies in the use of nationally representative survey data and the use of a broad sample size that represents the current situation in a very accurate  manner. We indeed, note that this study has a few limitations. First, causal inference is limited by the cross-sectional design of the study. Again, there may be other variables that may affect the perception of women that the NHIS helps pregnant women in  accessing health services that were not included in this study because of their unavailability in the dataset. Finally, the answer to the dependent variable–is NHIS helping pregnant women for health services or not–rely mainly on discretionary verbal responses of the women which did not consider a specific health service but looked at access to health serices in general.

## Conclusion

This present study has revealed a statistically significant association between NHIS subscription and women indicating that, it is helping pregnant women in accessing healthcare. In addition, ANC visits, age, educational level, wealth status and region influence women’s perception of the importance of NHIS in maternal healthcare access in Ghana. The findings from this study have highlighted the socio-demographic characteristics that influence women’s perception that subscribing to the NHIS is helping pregnant women in accessing health services. It is, therefore, imperative to encourage the subscription of NHIS. For example, the Maternal and Child Health Unit of the Ghana Health Service, in collaboration with non-governmental organisations dedicated to maternal and child health issues, could intensify efforts to educate pregnant women on the importance of NHIS subscription in maternal healthcare delivery.

## Data Availability

The dataset is freely available for download at the measure DHS website via: https://dhsprogram.com/data/dataset/Ghana_Standard-DHS_2014.cfm?flag=0

## Abbreviation

AOR: Adjusted Odds Ratio
CI: Confidence Interval;
GDHS: Ghana Demographic and Health Survey
DHS: Demographic and Health Survey
PHC: Population and Housing Census
NHIS: National Health Insurance Scheme
